# Fabrication of nanochannels with ladder nanostructure at the bottom using AFM nanoscratching method

**DOI:** 10.1186/1556-276X-9-212

**Published:** 2014-05-06

**Authors:** Yongda Yan, Yanquan Geng, Zhenjiang Hu, Xuesen Zhao, Bowen Yu, Qi Zhang

**Affiliations:** 1 Center for Precision Engineering, Harbin Institute of Technology, P.O. Box 413, Harbin, Heilongjiang 150001, People's Republic of China

**Keywords:** Atomic force microscopy, Nanochannel, Ladder nanostructure

## Abstract

This letter presents a novel atomic force microscopy (AFM)-based nanomanufacturing method combining the tip scanning with the high-precision stage movement to fabricate nanochannels with ladder nanostructure at the bottom by continuous scanning with a fixed scan size. Different structures can be obtained according to the matching relation of the tip feeding velocity and the precision stage moving velocity. This relationship was first studied in detail to achieve nanochannels with different ladder nanostructures at the bottom. Machining experiments were then performed to fabricate nanochannels on an aluminum alloy surface to demonstrate the capability of this AFM-based fabrication method presented in this study. Results show that the feed value and the tip orientation in the removing action play important roles in this method which has a significant effect on the machined surfaces. Finally, the capacity of this method to fabricate a large-scale nanochannel was also demonstrated. This method has the potential to advance the existing AFM tip-based nanomanufacturing technique of the formation these complex structures by increasing the removal speed, simplifying the processing procedure and achieving the large-scale nanofabrication.

## Background

Nowadays, the rapid development of microfluidic/nanofluidic systems has been seen in many applications such as fluid mixing [1,2], drug delivery [3], ion transporters [4], and DNA translocators [5]. The micro/nanochannels are the key components in the microfluidic/nanofluidic systems. Recently, more complex nanochannels (e.g., with some nanostructures at the bottom) are designed to study the influences on the flowing characteristic of fluid in the nano/microchannels [2]. The successful fabrication of these micro/nanochannels urgently needs to be solved. At present, the nanochannel fabrication methods mainly include focused ion beam milling [5], nanoimprint lithography [6], electron beam drilling [7], and wet chemical etching [8]. However, the complexity and/or cost of these methods greatly restrict the nanochannel fabrication, especially for the nanochannel with complex nanostructures at the bottom.

Since atomic force microscopy (AFM) was invented, the AFM tip-based nanomachining method had emerged as one of the essential technologies for nanostructure fabrication [9]. A lot of works have already been carried out to fabricate nanochannels on the surfaces of different kinds of materials using this method [10-15]. For example, Zhang et al. [13] presented an AFM-based high-rate tunable nanolithography technique to scratch nanochannels on PMMA surfaces. Kawasegi et al. [14] processed grooves and holes on the surface of the single-crystal silicon with an AFM diamond tip. Wang et al. [15] used the AFM-based repeated scratching method to obtain nanochannels on the silicon oxide surface. From these previous studies, it can be found that the AFM-based nanomechanical method is feasible for machining nanochannels. However, they were only able to fabricate V-shaped nanochannels or quadrate holes.

Recently, Arda Gozen et al. [16,17] developed a nanomilling system with an AFM tip as the small cutting tool to fabricate the three-dimensional and ladder-shaped nanostructures, which is similar to the traditional milling process. In our previous study [18], a width controllable millimeter-scale nanochannel array was also obtained by a modified AFM-based nanomachining system and the machined nanochannel showed a consistent depth. However, if a nanochannel with ladder structures at the bottom is needed, the stages must be controlled to reposition for secondary processing [16,18] or the normal load applied on the sample must be varied in the scratching process [19]. The reposition of the stage for secondary processing is less efficient especially for large-scale microstructures using the AFM tip-based nanofabrication method. In addition, the normal load must be controlled all the time according to the movement trajectory of the AFM tip during the whole machining process to obtain a nanochannel with ladder structure at the bottom, which is relatively complicated for the nanochannel fabrication.

Therefore, in this letter, we present a novel and easy AFM-based nanomanufacturing method combining the AFM internal tip scanning cycles with the high-precision stage movement to fabricate nanochannels with ladder nanostructure at the bottom. Using this method, a nanochannel with ladder nanostructure at the bottom can be achieved by continuous scanning with a fixed scan size. Different structures can be obtained according to the matching relation of the feeding velocity of the tip and the moving velocity of the precision stage. As such, this nanomachining method has the potential to advance the AFM tip-based nanomanufacturing by increasing the removal speed, simplifying the processing procedure, and achieving the large-scale nanofabrication.

## Methods

**Figure 1:**
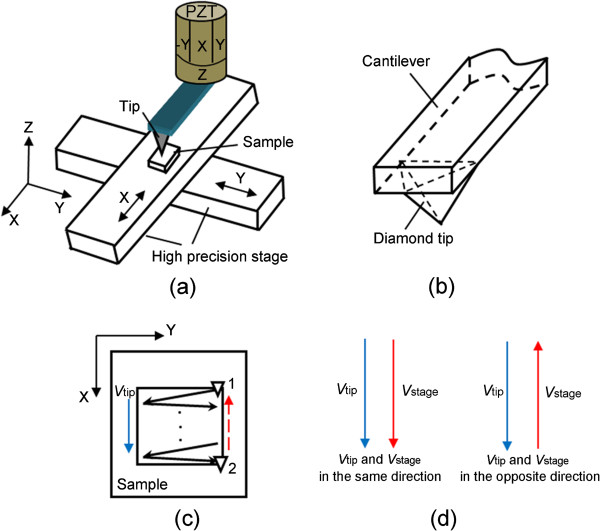
**Schematic of the nanochannel machining process. (a)** Schematic of the AFM-based nanomachining system. **(b)** The geometry of the diamond tip. **(c)** The tip scanning trajectory. **(d)** Two relative moving conditions.

Figure 1a shows the schematic of the modified AFM-based nanomachining system. The experimental setup mainly includes a commercial AFM (Q-Scope 250; Ambios Company, Santa Cruz, CA, USA) and two high-precision stages (M511.HD; PI Company, Eschbach, Germany). The detail information of the experimental facilities can be found in [18]. The AFM tip used for all nanoscratching tests is a diamond tip (DNISP; Veeco Instruments Inc., Plainview, NY, USA). This tip is a three-sided pyramidal diamond tip (Figure 1b) with a radius *R* of 85 nm evaluated by the blind reconstruction method [20]. The cantilever of the probe is made of stainless steel with a calibrated normal spring constant *K* of 174 N/m provided by the manufacturer. The sample used in the present study is aluminum alloy (2A12) machined by ultra-precision turning to obtain a surface roughness (*R*_a_) of 5 ± 2 nm. Because all scratching tests are carried out on the soft aluminum alloy, the rigid diamond tip exhibits negligible wear. After machining, the sample is imaged by scanning electron microscopy (SEM) immediately to observe the morphology of the chips formed in the scratching process. Before imaging by AFM, the machined sample is washed in alcohol solution ultrasonically for about 10 min to remove the chips. Then the fabricated region is scanned by a silicon nitride tip with a radius of less than 10 nm to obtain the 3-D topography of the nanochannels.

Based on this modified system, a novel and simple nanomachining method combining the scanning movement of AFM piezoceramics tube (PZT) with the rectilinear movement of the high-precision stage is realized. Utilizing this method, a nanochannel with ladder nanostructure at the bottom can be achieved by continuous scanning with a fixed scan size. The machining procedures are described as follows:

(1) The AFM system is set to contact mode, and the diamond AFM tip approaches the sample surface at a normal load which can make the tip press into the sample plastically. This normal load is used to control the depth of the nanochannels.

(2) The AFM is controlled to scan with a setting scan size regularly. As shown in Figure 1c, the AFM tip moves from the initial position (denoted by 1) to the end position (denoted by 2) to achieve one scanning cycle. After completing one scan, the AFM tip returns to the initial position (denoted by 1) to start another new scan operation. This process is repeated until the machining process is finished. Meanwhile, as shown in Figure 1a, the X direction high-precision stage moves at a low velocity (*V*_stage_) along the slow-scanning axis of the tip continuously. Two conditions can be generated: the stage moves in the same direction with the tip feeding velocity (*V*_tip_); the stage moves in the opposite direction to the tip feeding velocity (*V*_tip_). The scan size of AFM and the displacement moved by the high-precision stage are to control the width and the length of the nanochannel, respectively. Meanwhile, the dimension and the structure of the ladder machined at the bottom of the nanochannel are determined by the matching relationship between *V*_tip_ and *V*_stage_, which will be described in detail in the following sections.

(3) After one nanochannel is obtained, the AFM tip is lifted and the high-precision stage in the Y direction (shown in Figure 1a) is controlled to move to the next position. Another nanochannel can be machined with the same procedure. Thus, the channel arrays can be achieved.

Before machining, the scan size is set to a certain value, *L*_tip_. *f* is the scan rate and *s* is the number of line-scanning within one scanning process. Thus, the feeding velocity of the slow-scanning axis of the AFM tip (*V*_tip_ ) can be expressed by Equation 1. Moreover, the length of the nanochannel (*L*) is the distance traveled by the high-precision stage.

(1) $$V_{\mathrm{tip}}=f\cdot \frac{L_{\mathrm{tip}}}{s}$$

The two machining cases mentioned above are described as follows.

### Matching relations between *V*_tip_ and *V*_stage_ under the condition of the stage motion and the feed rate in the same direction

**Figure 2:**
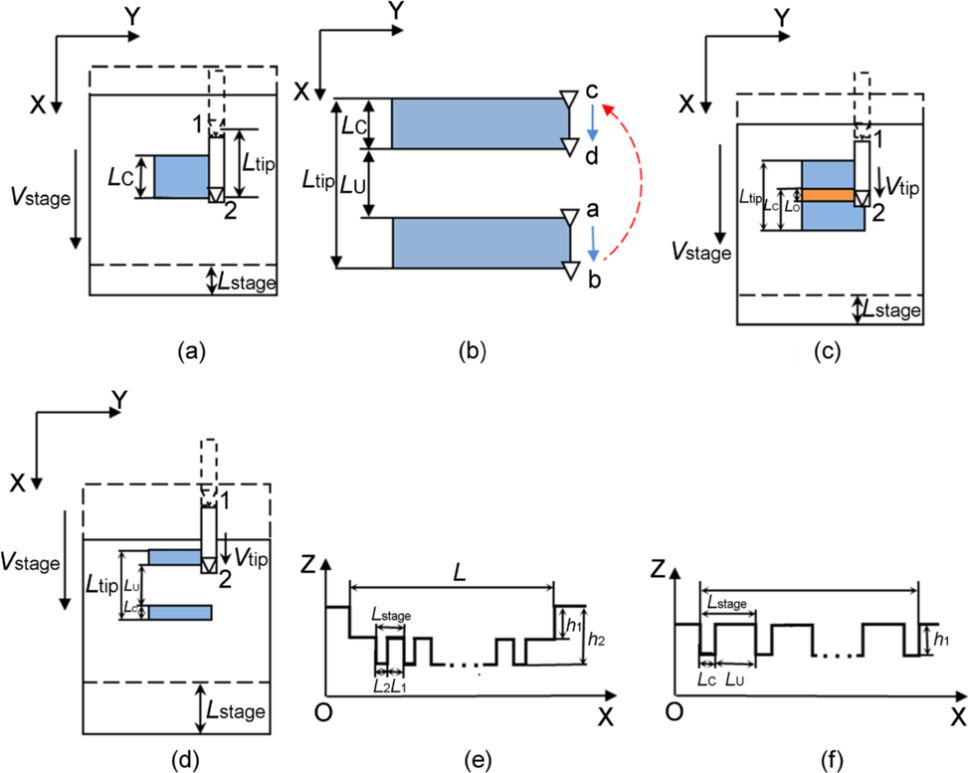
**Schematic of the nanochannel scratching with *V***_**stage **_**and *V***_**tip **_**in the same direction when *V***_**stage**_ **<** ***V***_**tip. **_**(a)** Schematic of the machining state after one AFM scanning cycle. **(b)** Schematic of the equivalent movement of AFM tip relative to the stage. Schematic of the machining state after two AFM scanning cycle **(c)** when *V*_stage_ < 0.5 *V*_tip_ and **(d)***V*_stage_ > 0.5 *V*_tip_. **(e)** Schematic of the cross section of the machined nanochannel with the typical condition of *N* = 0 when *V*_stage_ < 0.5*V*_tip_. **(f)** Schematic of the cross section of the machined nanochannel when *V*_stage_ > 0.5*V*_tip_.

**Figure 3:**
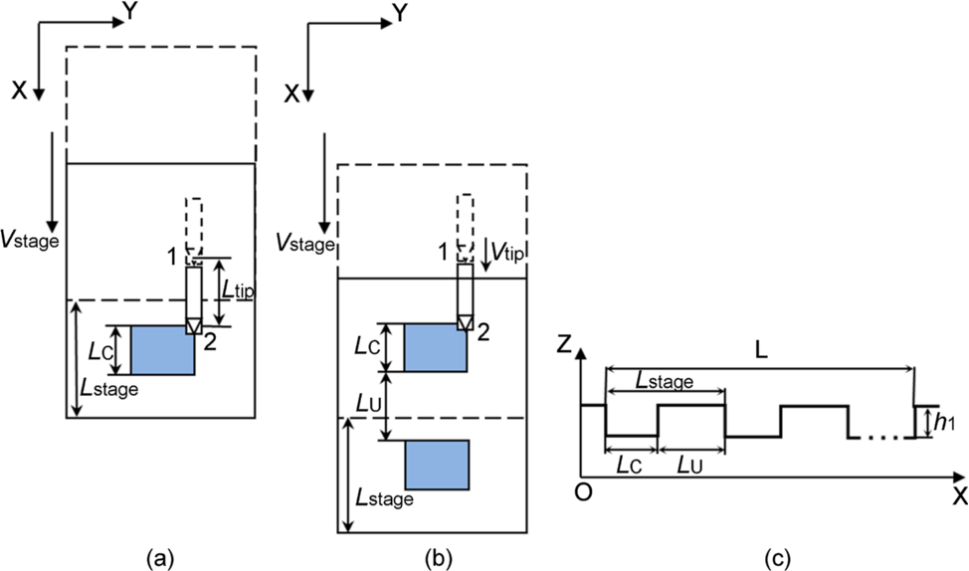
**Schematic of the nanochannel scratching with *V***_**stage **_**and *V***_**tip **_**in the same direction when *V***_**stage**_ **>** ***V***_**tip**_**.** Schematic of the machining state after **(a)** one and **(b)** two AFM scanning cycle. **(c)** Schematic of the cross section of the machined nanochannel.

In this condition as shown in Figures 2 and 3, the direction of the feeding velocity and the moving direction of the high-precision stage are both along the positive direction of *x* axis. The dotted and solid lines represent the previous and the following machining states, respectively. In terms of the velocity of the high-precision stage (*V*_stage_) comparing with *V*_tip_, the machining process in this situation can be divided into two scenarios as follows:

(1) When *V*_stage_ < *V*_tip_, the schematic of the machining process is shown in Figure 2. The tip scanning cycle and the high-precision stage movement are proceeding at the same time. As shown in Figure 2a, the tip moves from the start position 1 to the final position 2 to finish one tip scanning cycle and the blue region represents the machined area in one AFM scanning cycle. The length of the machined region in one AFM scanning cycle (*L*_C_) can be expressed by Equation 2. Then the tip returns to the initial position 1 to start the next scanning process. Considering the relative movement between the AFM tip and the stage, the equivalent movement of AFM tip relative to the stage is in the positive direction of *x* axis with a velocity of *V*_tip_ - *V*_stage_ as shown in Figure 2b. The path of the equivalent movement of the AFM tip is a → b → c → d. The tip moves from b to c caused by the tip finishing a scanning cycle to start a new cycle. The displacement from b to c is *L*_tip_ which is the scan size of the scanning. Thus, the two adjacent scratched regions are all in the area with the length of *L*_tip_. As shown in Figure 2b, when *L*_tip_ is equal to two times of *L*_C_, the adjacent scratched regions exactly connect with each other. Moreover, the length of the unmachined region (*L*_U_) is equal to 0. Thus, the critical value of *V*_stage_ is calculated to be half of *V*_tip_. Figure 2c,d shows the scratched states after two tip scanning cycles with the conditions of *V*_stage_ < 0.5*V*_tip_ and *V*_stage_ > 0.5*V*_tip_, respectively, which will be described in detail as follows:

(2) $$L_{C}=L_{\mathrm{tip}}-L_{\mathrm{stage}}=\left(V_{\mathrm{tip}}-V_{\mathrm{stage}}\right)\cdot \frac{s}{f}$$

As shown in Figure 2c, when *V*_stage_ is less than half of *V*_tip_, the two regions machined in the adjacent AFM scanning cycles have an overlapping machined region with a length (*L*_O_) expressed by Equation 3. If the *V*_stage_ is small to a certain value, the two adjacent overlapping machined regions also can overlap with each other. As shown in Equation 4, the ratio of *L*_O_ and *L*_stage_ can be expressed as an integer (*N*) plus a fraction (*a*). From the geometrical relationship, the lengths of the *N* + 1 and *N* + 2 times the overlapping machined region can be obtained by Equations 5 and 6, respectively. Through Equations 5 and 6, the period of the ladder nanostructure is calculated to be *L*_stage_. Figure 2e shows the schematic of the cross section of the machined groove with the typical condition of *N* = 0. *L*_1_ and *L*_2_ represent the lengths of the one and two times machined regions, respectively. *h*_1_ and *h*_2_ are the corresponding depths.

(3) $$L_{O}=L_{\mathrm{tip}}-2L_{\mathrm{stage}}=\left(V_{\mathrm{tip}}-2V_{\mathrm{stage}}\right)\cdot \frac{s}{f}$$

(4) $$\frac{L_{O}}{L_{\mathrm{stage}}}=N+a$$

(5) $$L_{N+1}=\left(1-a\right)\cdot V_{\mathrm{stage}}\cdot \frac{s}{f}$$

(6) $$L_{N+2}=a\cdot V_{\mathrm{stage}}\cdot \frac{s}{f}$$

As shown in Figure 2d, when *V*_stage_ is larger than half of *V*_tip_, the two regions machined in the adjacent AFM scanning cycles are nonoverlapping, which can cause a length of the unmachined region (*L*_U_) expressed by Equation 7. Through Equations 2 and 7, the period of the ladder nanostructure is also calculated to be *L*_stage_. Figure 2f shows the schematic of the cross section of the machined groove in this condition. *h*_1_ represents one-time machined depth.

(7) $$L_{U}=2L_{\mathrm{stage}}-L_{\mathrm{tip}}=\left(2V_{\mathrm{stage}}-V_{\mathrm{tip}}\right)\cdot \frac{s}{f}$$

The real pitch in scratching (Δ) in these two conditions mentioned above can be obtained by Equation 8:

(8) $$\Delta =\left(V_{\mathrm{tip}}-V_{\mathrm{stage}}\right)\cdot \frac{1}{2f}$$

(2) When *V*_stage_ > *V*_tip_, as shown in Figure 3, the scratched state is different from the condition shown in Figure 2. Figure 3a,b shows the machined states of after one and two tip scanning cycles, respectively. By considering the geometric relationship, as shown in Figure 3b, *L*_C_, *L*_U_, and Δ can be obtained by Equations 9, 10, and 11, respectively. The length of the unmachined region (*L*_U_) only depends on the displacement of the AFM tip in one scanning cycle. From Equations 9 and 10, the period of the ladder nanostructure is calculated to be *L*_stage_. Figure 3c shows the schematic of the cross section of the machined groove in this condition. *h*_1_ represents the one-time machined depth.

(9) $$L_{C}=L_{\mathrm{stage}}-L_{\mathrm{tip}}=\left(V_{\mathrm{stage}}-V_{\mathrm{tip}}\right)\cdot \frac{s}{f}$$

(10) $$L_{U}=L_{\mathrm{tip}}=V_{\mathrm{tip}}\cdot \frac{s}{f}$$

(11) $$\Delta =\left(V_{\mathrm{stage}}-V_{\mathrm{tip}}\right)\cdot \frac{1}{2f}$$

### Matching relations between *V*_tip_ and *V*_stage_ under the condition of the stage motion and the feed rate in the opposite direction

**Figure 4:**
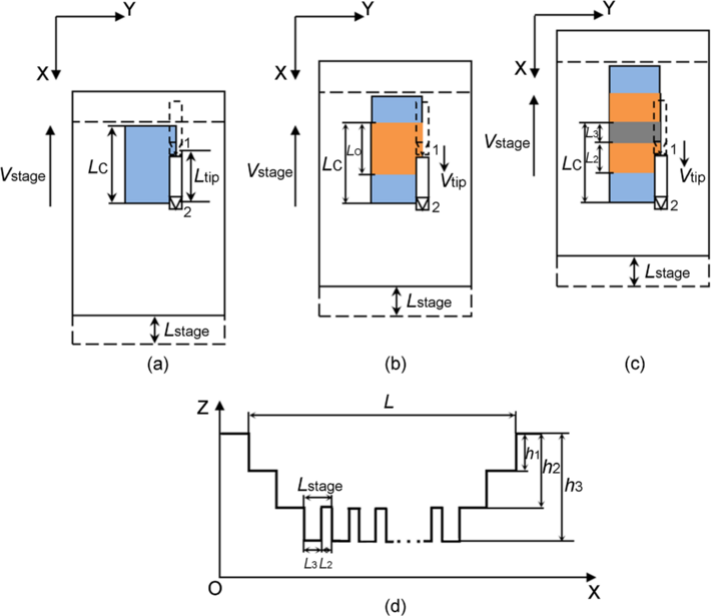
**Schematic of the nanochannel scratching with *V***_**stage **_**and *V***_**tip **_**in the opposite direction when *V***_**stage**_ **<** ***V***_**tip.**_ Schematic of the machining state after **(a)** one, **(b)** two, and **(c)** three AFM scanning cycles. **(d)** Schematic of the cross section of the machined nanochannel with the typical condition of *N* = 1.

**Figure 5:**
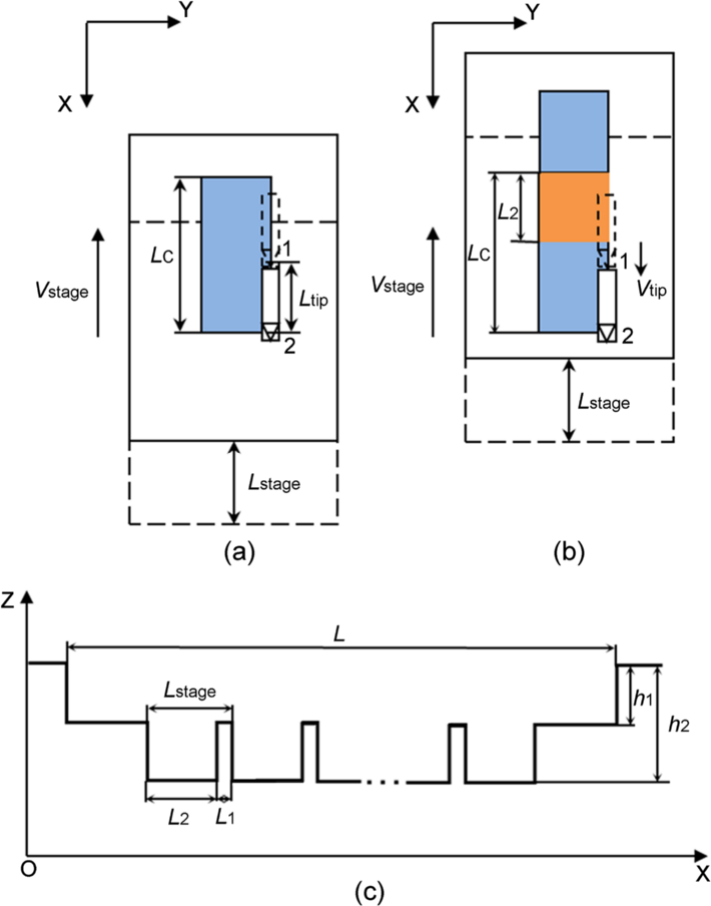
**Schematic of the nanochannel scratching with *V***_**stage **_**and *V***_**tip **_**in the opposite direction when *V***_**stage**_ **>** ***V***_**tip**_**.** Schematic of the machining state after **(a)** one and **(b)** two AFM scanning cycle. **(c)** Schematic of the cross section of the machined nanochannel.

In this condition as shown in Figures 4 and 5, the feeding direction is along the positive direction of *x* axis, and the moving direction of the high-precision stage is along the negative direction of *x* axis. As the moving direction of the high-precision stage is contrary to the tip feeding direction, the displacement of the tip relative to the sample is in the positive direction of *x* axis. Similar to the stage motion and the feed rate in the same direction scratching process, the machining process with the opposite direction is also divided into the following conditions according to the high-precision stage velocity:

(1) When *V*_stage_ < *V*_tip_, Figure 4a,b,c shows the schematic of the fabricated nanochannel after one, two, and three tip scanning cycles, respectively. The blue block is the fabricated region in one tip scanning cycle with a length (*L*_C_) expressed by Equation 12, shown in Figure 4a. The yellow block, shown in Figure 4b, is the overlapping region of the two adjacent fabricated regions with a larger depth. Due to the *L*_stage_ smaller than the *L*_tip_, the two adjacent overlapping machined regions can also be overlapped with each other (gray region with a length (*L*_O_)), as shown in Figure 4c. As shown in Equation 13, the ratio of *L*_tip_ and *L*_stage_ can be expressed as an integer (*N*) plus a fraction (*a*). By considering the geometric relationship, the lengths of the *N* + 1 and *N* + 2 times overlapping machined region can be obtained by Equations 14 and 15, respectively. From Equations 14 and 15, the period of the ladder nanostructure is calculated to be *L*_stage_. Figure 4d shows the schematic of the cross section of the machined groove in this condition with the typical condition of *N* = 1. *L*_2_ and *L*_3_ represent the lengths of the two and three times machined regions, respectively. *h*_2_ and *h*_3_ are the corresponding depths. *h*_1_ represents the depth of one-time machined region. Moreover, the real pitch in scratching (Δ) in this condition can be obtained by Equation 16:

(12) $$L_{c}=L_{\mathrm{stage}}+L_{\mathrm{tip}}=\left(V_{\mathrm{stage}}+V_{\mathrm{tip}}\right)\cdot \frac{s}{f}$$

(13) $$\frac{L_{O}}{L_{\mathrm{stage}}}=\frac{L_{\mathrm{tip}}}{L_{\mathrm{stage}}}=N+a\;\left(N\;\mathrm{is}\;\mathrm{integer}\right)$$

(14) $$L_{N+1}=\left(V_{\mathrm{stage}}-a.V_{\mathrm{stage}}\right)\cdot \frac{s}{f}$$

(15) $$L_{N+2}=a\cdot V_{\mathrm{stage}}\cdot \frac{s}{f}$$

(16) $$\Delta =\left(V_{\mathrm{stage}}+V_{\mathrm{tip}}\right)\cdot \frac{1}{2f}$$

(2) When *V*_stage_ > *V*_tip_, similar to the condition described in part (1), the blue block which is the fabricated region for one scanning cycle with a length (*L*_C_) can also be expressed by Equation 12, shown in Figure 5a. The yellow block, shown in Figure 5b, is the overlapping region of the two fabricated regions with a larger depth. Due to the *V*_stage_ larger than the *V*_tip_, the two adjacent overlapping machined regions cannot be overlapped with each other. As shown in Figure 5c, the lengths of one (*L*_1_) and two times (*L*_2_) overlapping machined regions can be obtained by Equations 17 and 18, respectively, and *h*_1_ and *h*_2_ are the corresponding depths. From Equations 17 and 18, the period of the ladder nanostructure is also calculated to be *L*_stage_. Figure 5c shows the schematic of the cross section of the machined groove in this condition. The real pitch in scratching (Δ) in this condition maintained above also can be obtained by Equation 16.

(17) $$L_{1}=L_{\mathrm{stage}}-L_{\mathrm{tip}}=\left(V_{\mathrm{stage}}-V_{\mathrm{tip}}\right)\cdot \frac{s}{f}$$

(18) $$L_{2}=L_{\mathrm{tip}}=V_{\mathrm{tip}}\cdot \frac{s}{f}$$

To demonstrate the capability of the AFM-based fabrication method presented in this study, five channels with different machining parameters corresponding to the conditions mentioned above were created on the aluminum alloy sample. The scan size (*L*_tip_), scan rate of the AFM (*f*), and the number of line-scanning within one scanning process (*s*) are set to 10 μm, 4 Hz, and 300, respectively, for all scratching tests. Thus, the feed velocity of the AFM tip *V*_tip_ is calculated to be 133.3 nm/s using Equation 1. The machining results are described and analyzed in detail in Section ‘Results and discussion’.

## Results and discussion

**Figure 6:**
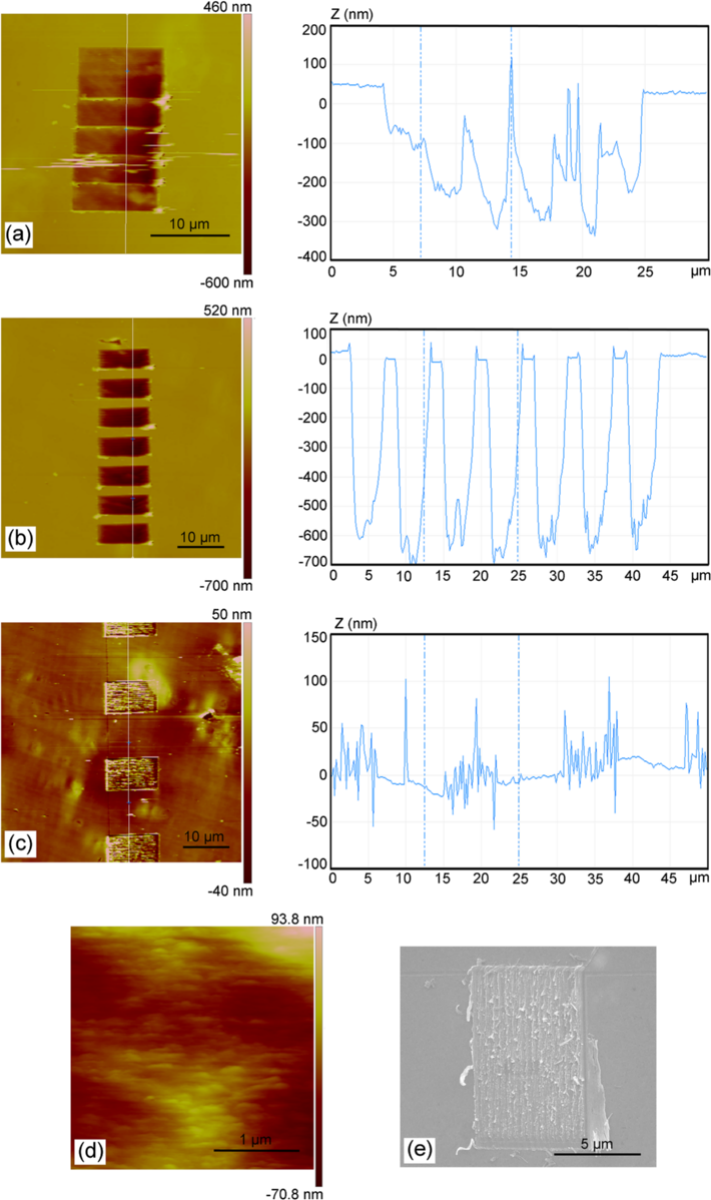
**Nanochannels scratched with *V***_**stage **_**and *V***_**tip **_**in the same direction. (a-c)** The AFM images of the machined nanochannel with different *V*_stage_. **(d)** The local AFM image of the nanochannel bottom machined with *V*_stage_ of 50 nm/s. **(e)** The SEM image of the nanochannel machined with *V*_stage_ of 200 nm/s.

**Figure 7:**
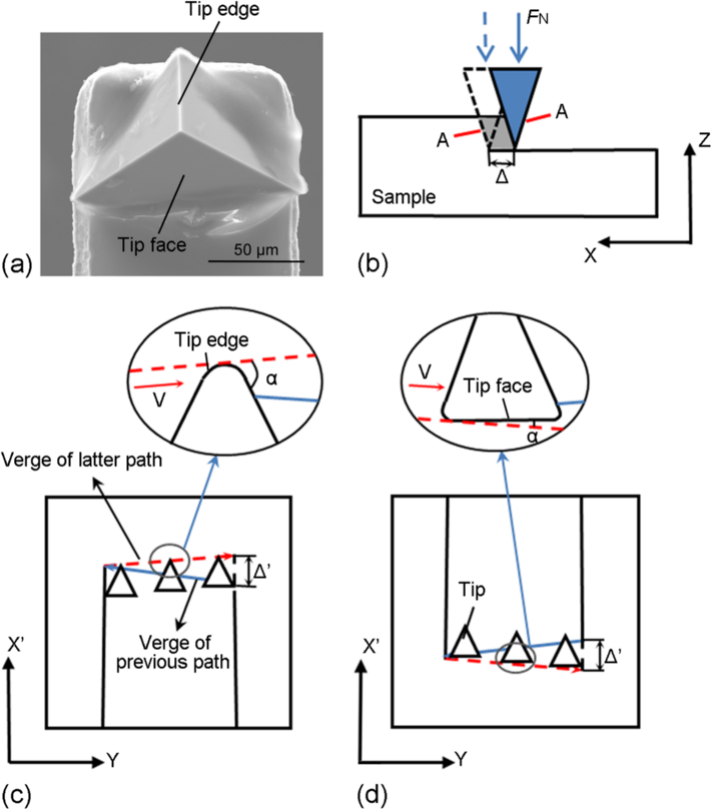
**Schematic of material removal mechanisms by an AFM tip. (a)** The SEM image of the diamond AFM tip. **(b)** The front view of the nanochannel fabrication process. The A-A cross-section indicated in Figure 7**(b)** with the displacement of the tip relative to the sample during one scanning process in the **(c)** positive and **(d)** negative direction of *x* axis.

Figure 6 shows the AFM and SEM images of the nanochannels scratched with the stage motion and the feed rate in the same direction. As shown in Figure 6a, the nanochannel machined with the stage velocity *V*_stage_ of 50 nm/s and the normal load of 36.06 μN has two-ladder structure, which agrees well with the condition shown in Figure 2c discussed in the part (1) of Section 3.1 (*V*_stage_ < 0.5*V*_tip_). However, the fluctuation of the channel bottom is very large. Due to *V*_tip_ larger than *V*_stage_, the displacement of the tip relative to the sample in one scanning process is in the positive direction of *x* axis shown in Figure 2a. As shown in Figure 7a which is the SEM image of the AFM diamond tip, the edge and the face of the tip can be observed clearly. Figure 7b shows the front view of the nanochannel fabrication process, and Figure 7c shows the A-A cross section indicated in Figure 7b, which represents the condition with the displacement of the tip relative to the sample in one scanning process in the positive direction of *x* axis. Δ′ and *x*′ axis, shown in Figure 7c, are defined as the projections of the feed of the tip (Δ) and *x* axis in the A-A cross section. In addition, *α* is the attack angle between the tip and the sample surface which can be used to determine the removal mechanisms of the materials. Thus, considering the geometry of the AFM tip shown in Figure 7c, the edge of the AFM tip plays a main role in the scratching test. For increasing *α*, three removal mechanisms have been proposed: plowing, wedge formation, and cutting [21]. For AFM diamond-tip-based nanomachining, if the attack angle is larger than a certain value (75° in [22]), cutting is the dominant mechanism. Using Equation 11, the real pitch in scratching is calculated to be 10 nm. Although the edge of the AFM tip plays a main role in the scratching test, machining with such small feed leads to a small attack angle resulting in plowing machining state, no obvious trace of the cutting tool left at the bottom of the channel (see Figure 6d) and a relatively rough surface, which agrees well with previous study [18]. The effective removal of the material mainly in the form of chips, rather than only piled up by plowing, is one of the crucial premises of the nanomachining process [17]. Therefore, such small feed is unsuitable for machining nanochannels. Similarly, the nanochannel shown in Figure 6b does not have a smooth bottom with the stage velocity (*V*_stage_) of 80 nm/s (the condition shown in Figure 2f: 0.5 *V*_tip_ < *V*_stage_ < *V*_tip_) and the normal load of 72.12 μN. The real pitch (Δ) is 6 nm obtained by Equation 11. Due to the real pitch (Δ) in scratching expressed in Equation 11 achieved by the *V*_tip_ minus *V*_stage_, the feed of the machining can hardly reach the value as large as to ensure the cutting state playing a main role in the scratching test. Moreover, the period of the ladder shown in Figure 6b is approximately 6.260 μm which is 260 nm larger than the calculated value of *L*_stage_ (6 μm). This is because the time of the AFM tip returning to the initial position (1 shown in Figure 1c) to start the next scanning cycle is about 3 s. In this period of time (*t*), the stage is still moving for a displacement of *V*_stage_*t*. Thus, the experimental period of the ladder structure has a displacement of *V*_stage_*t* larger than the theoretical equations devised. Simultaneously, the displacement caused by this interval time should be added into the length of the unmachined region. The channel in Figure 6c is machined with the stage velocity of 200 nm/s (the condition shown in Figure 3c: *V*_tip_ < *V*_stage_) and the normal load of 72.12 μN. From the cross section of the channel shown in Figure 6c, it can be observed that there is almost no scratched depth of the channel. Figure 6e shows the SEM image of the scratched region under this condition. From the SEM image, lots of larger burrs remained on both sides of the trace of the AFM tip. In this condition, due to *V*_stage_ larger than *V*_tip_, the displacement of the tip relative to the sample is in the negative direction of *x* axis shown in Figure 3a. Figure 7d shows the A-A cross section indicated in Figure 7b with the displacement of the tip relative to the sample in one scanning process in the negative direction of *x* axis. As the real pitch (Δ) in scratching is much smaller than the width of the machined nanochannel, the attack angle *α* is very small, which is closed to 0. From Figure 6e, large burrs can be observed on the right side of the nanochannel and it can be indicated that the material of the sample must be extruded by the face of the tip. Thus, plowing is the dominant mechanism in this condition and the materials cannot be effectively removed, that is, this condition may be unsuitable for the nanochannel fabrication in the present study.

**Figure 8:**
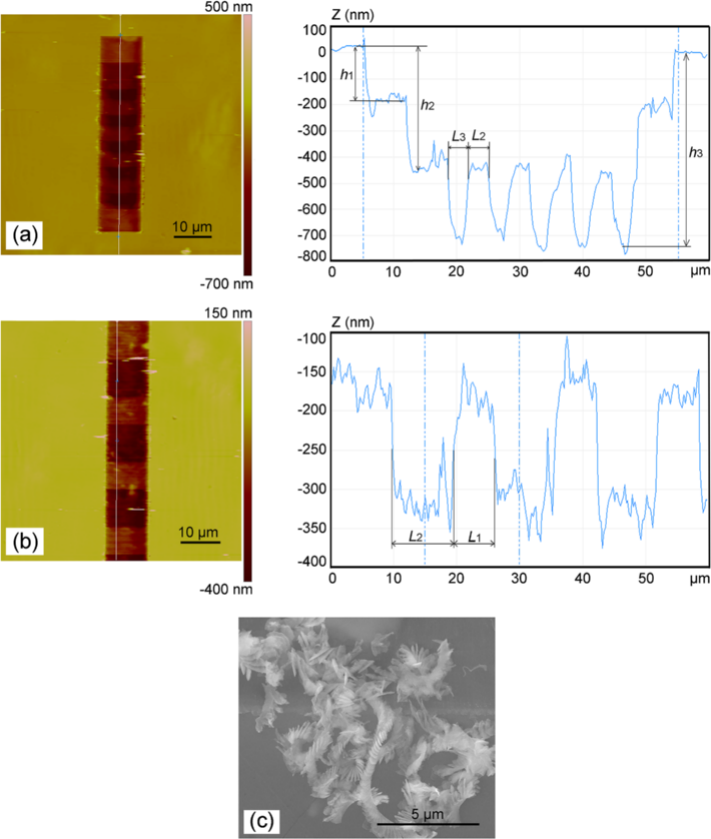
**Nanochannels scratched with *V***_**stage **_**and *V***_**tip **_**in the opposite direction. (a-b)** The AFM images of the machined nanochannel with different *V*_stage_. **(c)** The SEM image of the chips of the machined nanochannel.

Figure 8 shows the AFM and SEM images of the nanochannels scratched with the stage motion and the feed rate in the opposite direction. Figure 8a,b shows the AFM images of the nanochannel with the stage velocities of 80 nm/s (the condition shown in Figure 4d: *V*_stage_ < *V*_tip_) and 200 nm/s (the condition shown in Figure 5c: *V*_tip_ < *V*_stage_), respectively. For each case, the normal load is set to 72.12 μN. In Figure 8a, *L*_2_ and *L*_3_ are approximately 2.588 and 3.720 μm, respectively. The corresponding depths *h*_1_, *h*_2_, and *h*_3_ are 203, 440, and 688 nm, respectively. *L*_3_ is about 255 nm less than the value obtained by Equation 15 (3.975 μm). In Figure 7b, *L*_1_ and *L*_2_ are approximately 6.142 and 9.372 μm, respectively. The corresponding depths *h*_1_ and *h*_2_ are 241 and 395 nm, respectively. *L*_2_ is about 638 nm less than the value obtained by Equation 18 (10 μm). Similar to the discussion above, by considering the time of the AFM tip returning to the initial position (1 shown in Figure 1c) to start the next scanning cycle (*t*) in both conditions, the periods of the ladder nanostructure have a value of *V*_stage_*t* larger than *L*_stage_ that resulted from the continuous motion of the stage in this period of time. Meanwhile, the lengths of the overlapping region with the largest depth in the nanochannels have a length of *V*_stage_*t* less than the calculated values obtained by Equations 15 and 18. The real pitches (Δ) of these two conditions are 27 and 42 nm, respectively, obtained by Equation 16. Moreover, the displacement of the tip relative to the sample in one scanning process is in the positive direction of *x* axis as shown in Figures 4a and 5a. From Figure 7c, it can be indicated that the edge of the tip plays a main role in the scratching test in these cases. Figure 8c shows the SEM image of the cutting chips after machining. It is indicated that within these feeds, materials are mainly removed by the cutting state with a relatively large attack angle (*α*), which is able to effectively remove material, and nanochannels with good quality can be achieved in these conditions.

**Figure 9:**
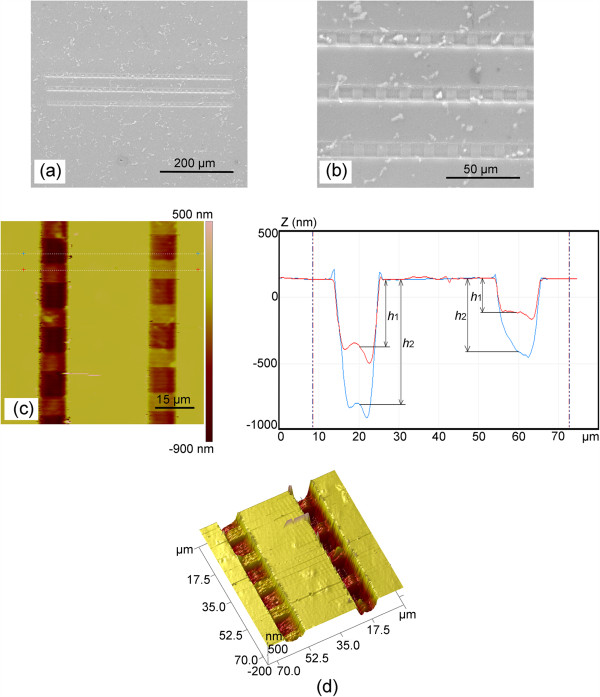
**Large-scale nanochannels array.** The **(a) **whole and **(b)** local SEM images of the machined nanochannel array. **(c)** The local AFM image of the machined nanochannel array. **(d)** 3D AFM image of the machined nanochannel array.

To show the capability of this method in creating large-scale channels with the ladder nanostructures, a set of nanochannels are fabricated on the sample. Figure 9a,b shows the SEM images of the overall and local part of the fabricated channels with the length of 500 μm and the width of 10 μm. As shown in Figure 9a, the above two channels and the underneath one are machined with the normal load of 95.96 and 194.24 μN, respectively. *V*_tip_ is 133.3 nm/s, and *V*_stage_ is set to 200 nm/s (the condition shown in Figure 5c: *V*_tip_ < *V*_stage_). Figure 9c,d shows the 2D and 3D AFM images of the local part of the fabricated channels. The ladder nanostructures can be observed at the bottom of the nanochannels. In Figure 9c, *L*_1_ and *L*_2_ are approximately 6.141 and 9.417 μm, respectively. Meanwhile, the period of the ladder nanostructure is approximately 15.558 μm. The corresponding depths *h*_1_ and *h*_2_ are 320 and 619 nm, respectively, with the normal load of 95.96 μN. With the normal load of 194.24 μN, the corresponding depths *h*_1_ and *h*_2_ are 648 and 1,081 nm, respectively.

## Conclusions

In summary, this letter presents an AFM-based nanomachining method to fabricate nanochannels with ladder nanostructure at the bottom. The ladder nanostructures can be obtained by continuous scanning of the AFM tip according to the matching relation of the velocities of the tip feeding and the precision stage moving. With the high-precision stage moving in the same direction with the tip feeding velocity, the tip feed can hardly reach as large as the value to ensure the cutting state playing a main role in the scratching test. Simultaneously, in this condition, when the stage moving velocity is larger than the tip feeding velocity, the nanochannel cannot be obtained due to extremely small attack angle in the machining process and the materials cannot be effectively removed. On the contrary, when the stage moves opposite to the feeding direction, an appropriate feed value can be easily achieved. Moreover, the edge of the tip plays an important role in the scratching tests. The materials are mainly removed by the cutting state in this condition resulting in good surface quality. The perfect nanochannel with ladder nanostructure at the bottom can be obtained under this condition. Moreover, a large scale of the length of 500 μm and the width of 10 μm of such kind of nanochannel is machined successfully using this novel method.

It is expected that this AFM-based nanomachining method will yield more complex structures through controlling the movement of the PZT of the AFM. In addition, the future work will enable to identify the optimal nanomachining parameters.

## Abbreviations

AFM: atomic force microscopy; SEM: scanning electron microscopy; PZT: piezoceramics tube; 2D: two-dimensional; 3D: three-dimensional.

## Competing interests

The authors declare that they have no competing interests.

## Authors’ contributions

YDY and YQG carried out the design and drafted the manuscript. XSZ and ZJH participated in the experiments. BWY and QZ assisted with the optimization and proofed the manuscript. All authors read and approved the final manuscript.
